# Diet in Disease

**Published:** 1893-04-08

**Authors:** Ernest Hart

**Affiliations:** Bachelier-ès-Sciences-es-Lettres (rèstreint), formerly Student of the Faculty of Medicine of Paris, and of the London School of Medicine for Women


### Diet in Disease.

By Mrs. Ernest Hart, Bachelier-ès-Sciences-es-Lettres (rèstreint), formerly Student of the Faculty of Medicine of Paris, and of the London School of Medicine for Women.

XIX.—INDIGESTION (*continued*).

**The Symptoms of Indigestion.**—In those in whom the process of digestion is normal, eating only gives a sensation of satisfaction. The food passes from the stomach into the duodenum without exciting any uncomfortable feelings, and the person goes about his occupations untroubled by the cares of the body till hunger tells him it is time to eat again. Not so with the dyspeptic. Eating gives at first the sense of satisfaction, but this is soon followed by a feeling of distress at the pit of the stomach. The waist seems and is unnaturally distended, eructations of flatulence take place, the stomach feels sore inside, pain spreads to the region under the shoulder blades, the intelligence becomes dulled, the temper irritable, the spirits depressed, and there is a tendency to drowsiness, the indulgence in which is at once the temptation to and the refuge of the dyspeptic. That a person is suffering "only from indigestion" is often thought to be a reason for expressing no sympathy with his malady; but, in my opinion, there is no condition which is more worthy of our pity, and that, in fact, a severe illness is much more endurable than the daily constant miseries of the dyspeptic. The healthy can scarcely realize what he suffers, the discomfort which does not amount to pain, the depression which does not reach melancholia, the irritability of which the manifestation makes him shunned by his friends. While he wants to dine he dreads to eat; when he longs to be cheerful he feels in the depths of low spirits; when he wishes to be kind he cannot help being cross. He is an unfortunate, to be pitied, to be borne with patiently and to be helped; but first of all he must help himself. He must make his own condition, his tiresome, contradictory, ill-regulated stomach his study, and must discover what to eat and what to avoid, and having discovered the rules by which to govern himself he must abide by them. Doctors to whom dyspeptics go for treatment and advice are fond of giving their patients written or printed lists of the things they may eat and not eat, the times for meals, &c. This rule of thumb may answer fairly well with a mass of people, but it is scarcely intelligent or scientific. These lists, and even the prescriptions, are handed on from one dyspeptic to another in the hope that the talisman may act without the payment of the standard fee. The results of this haphazard method of treating a most complicated malady would afford comic reading if they could be collected, and would doubtless form material for many miraculous cures. Asking once for something requiring immediate attention to be made up for me at a well-known chemist's, I was informed that it could not possibly be done that day, as the Countess of —— was going away into the country, and had sent all her prescriptions to be made up—they being all carefully preserved for country dispensation to her poor neighbours and dependents. I cannot resist the temptation to tell the following story, illustrating the way in which dietetic rules for the treatment of indigestion are handed on and looked upon as infallible specifics. Four men unknown to each other once met at the common table of a country inn. They all paid evident attention to what they ate. One refused the soup, and remarked, "Sir A. B. forbids soup at dinner"; another objected to drink anything, saying, "Sir A. B. advises that the meals should be taken dry." A third rejected the entrées and sweets, and sighed pathetically, "All kickshaws are tabooed by Sir A. B." The fourth man, however, was observed to eat steadily through the dinner, and to partake of all the good things with evident relish. "Sir," at last said one of his companions, "you do not seem to follow the dicta of Sir A. B." "No," was the genial reply of the man who had enjoyed his dinner, "for I am Sir A. B." I once had the audacity to tell this story to the great physician indicated, and no one's amusement could have been greater or his laughter more hearty.

**To Treat Dyspepsia Dietetically** there are certain broad principles to be followed. First, the bowels should be regulated with care, watchfulness, and intelligence. Both constipation and diarrhœa should be avoided. It is of the utmost importance that, on the one hand, the digested food should not lodge or stagnate in the intestines, there undergoing fermentative changes and causing flatulence and distress; nor on the other, should it be hurried through the intestines without the opportunity for proper assimilation. Constipation is sometimes caused by the patient taking food which is too easily digested, and the peristaltic action of the intestines is hence not excited by the presence of undigested morsels. In these cases vegetables will often effect a cure. A glass of water taken on rising will have in many cases both a tonic and aperient effect. Chronic diarrhœa can be often checked by taking the most easily digested food and raw meat juice, the preparation of which was described in the article on invalid foods. It is incorrect to think that constipation and diarrhœa can only be cured by pills and draughts; a careful dietary can do more to establish a healthy condition of the intestinal mucous membrane than the use of drugs.

In cases of atonic dyspepsia caused by want of nervous tone the meals should be small and frequent if they can be well borne. The period of time between meals necessary to digest each meal properly can only be ascertained in the case of each patient individually by experiment. It is, however, the greatest possible mistake for a dyspeptic to force himself to eat. If he is not hungry it is probably because gastric juice has not been secreted in sufficient quantity to enable him to digest a meal. An attack of indigestion will therefore probably follow if food be taken. The reasonable thing to do is not to oblige the patient to eat, but to give him the material out of which the stomach can manufacture the pepsine required to perform digestion. This can be done by giving a small cupful of beef-tea half an hour before the meal. The beef-tea is rapidly absorbed, a stimulus is given to secretion, and the gastric juice is produced and poured out in time to digest the subsequent meal. This treatment of indigestion was discovered by the physiologist Schiff. It is too little known and practised. In all cases of indigestion the meals should be simple; that is, composed of few dishes, and one or two things only should be eaten at the same time. Thus, a dyspeptic may, perhaps, eat a cut of roast beef with comfort; but if he heaps his plate with potatoes, green vegetables and Yorkshire pudding, and eats them altogether, he will infallibly suffer from flatulence and indigestion. It would be better for him to eat his vegetables at one meal and his beef at another. It is better also to drink between meals and not at meals. To make this a habit is, in some cases, alone sufficient to cure obstinate dyspepsia. Pastry mysterious concoctions of preserves and flour, rich, greasy and highly spiced and flavoured foods should, as a rule, be avoided by the dyspeptic, though it is important that the food should be well cooked and daintily served, and that variety should be studied. "By variety," says Dr. King Chambers, "is meant not a great number of dishes at once, which is confusing and oppressive, and destructive of the object aimed at, but a frequent (why not daily ?) difference in the principal dish, to which the few other dishes are harmonised. Some of the most appetising dinners one has ever eaten have really consisted of one article, novel and unexpected. The famous Mrs. Poyser sagely remarked that a man's stomach likes to be surprised, and no surprise is possible if the same monotonous superfluity is repeated day by day."

In the intelligent combination of simplicity with variety, and of good cooking with both, lies the secret of the power to relieve much of the discomfort of the dyspeptic.

**Whether alcohol** should be taken or not is a subject again for experience. In many persons whose dyspepsia is the result of sedentary life and too close an application to anxious work in close rooms, a small amount of alcohol with meals undoubtedly promotes digestion; if, however, it causes flushing of the face and throbbing of the arteries it should not be taken. Good whiskey or brandy well diluted is often better borne than fermented wines. Tea acts on some dyspeptics like poison, producing a sense of weight in the chest, palpitation of the heart, and nervous excitement. If taken weak,and if the tea leaves be removed three minutes after the tea is made, it can be digested and has a refreshing and invigorating effect. Sugar should be taken sparingly by persons over forty; vegetables should be cooked well and in a variety of ways.

Probably the best of all cures for dyspepsia is **fresh air.** I am acquainted with chronic and constantly suffering dyspeptics who lose their dyspepsia as if by magic on going on board ship and sailing across the ocean. High, dry, bracing, sunny climates are the best, in which outdoor exercises such as riding and golfing can be enjoyed. Cheerful society should be sought, and even "frivolous conversation" is recommended by Dr. King Chambers at meals. Perhaps our forefathers had better judgment than ourselves when they enjoyed the jokes of the jester after a banquet instead of listening to the solemn perorations of the speechmakers. To consider his dyspepsia scientifically and philosophically, to study it, to lay down rules for his own guidance, and then as far as possible to forget his malady, should be the aim and practice of the dyspeptic.

